# Improving productive performance, immunity, and health status of growing rabbits by using honey bee venom (*Apis mellifera*)

**DOI:** 10.3389/fvets.2023.1234675

**Published:** 2023-09-28

**Authors:** Alaa E. Elkomy, Tarek A. Sadaka, Saber S. Hassan, Omnia Shawky, Mohamed E. El-Speiy, Mohamed El-Beshkar, Mohammad A. M. Wadaan, Hatem M. El-Tahan, Sungbo Cho, In Ho Kim, Hossam M. El-Tahan

**Affiliations:** ^1^Livestock Research Department, Arid Lands Cultivation Research Institute, City of Scientific Research and Technology Applications (SRTA-City), New Borg El Arab, Egypt; ^2^Faculty of Desert and Environmental Agriculture, Matrouh University, Mersa Matruh, Egypt; ^3^Animal Production Research Institute (APRI), Agriculture Research Center (ARC), Ministry of Agriculture, Dokki, Egypt; ^4^Department of Animal and Poultry Production, Faculty of Agriculture, Damanhour University, Damanhour, Egypt; ^5^El-Beshkar Manhal for Bee Breeding and Bee Products, Cairo, Egypt; ^6^Department of Zoology, College of Science, King Saud University, Riyadh, Saudi Arabia; ^7^Animal Resource and Science Department, Dankook University, Cheonan, Republic of Korea

**Keywords:** bee venom, growing rabbit, redox status, biochemical blood constituent, cecal microbial count

## Abstract

To investigate the effect of bee venom (BV) as a natural growth promotor on growing rabbits as an alternative to antibiotics, sixty 35-day-old Californian male rabbits with an average body weight of 584 ± 9 gm were randomly divided into five equal groups as follows: The 2nd group received drinking water supplied with 10 mg Oxytetracycline (OXT), while the 3rd, 4th, and 5th groups received 2, 4 and 8 mg bee venom (BV)/kg body weight/day in drinking water, and the first group was served as a control group. The growth performance features were positively impacted by adding BV (*p* ≤ 0.01) compared to the control, whereas LBW and BWG increased and FI reduced. Significantly improved carcass characteristics (*p* ≤ 0.01) as a result of the BV supplementation. Blood characteristics showed a significant reduction (*p* ≤ 0.01) in liver enzyme activities and Cholesterol, Triglycerides, and Low-density lipoproteins Cholesterol (LDL) as affected by BV treatment; inversely, total protein and globulin were significantly increased (*p* ≤ 0.01). Similarly, BV had a positive effect (*p* ≤ 0.01) on anti-oxidant status (Total anti-oxidant capacity (TAC), Glutathione peroxidase (GPx), superoxide dismutase (SOD) and catalase (CAT)). In contrast, the lipid peroxidation biomarker (Malondialdehyde (MDA)) was significantly decreased. The immunoglobulin (IgG and IgM) was significantly increased (*p* ≤ 0.01) by BV treatment. There was a positive effect of low BV levels on decreasing both cecum TBC and pathogenic bacterial count (*Salmonella* spp., *E.coli* spp., *Proteus* spp., and *Clostridia* spp.) that was significant (*p* ≤ 0.01). In conclusion, BV can be a natural growth promoter to enhance growth performance traits, immunological and anti-oxidative responses, and reduce pathogenic bacteria in the hindgut of growing rabbits.

## 1. Introduction

Animal welfare, ecological sustainability, and controlled usage of medical pharmaceuticals are all objectives for producing healthy products devoid of chemical residues or pharmaceutical drugs (1). Recently, along with implementing antibiotics bans in many countries, several studies have been conducted to search for alternatives to antibiotics in the livestock industry (2) due to increased interest in human nutrition (3).

Since ancient times, honey bee venom (BV) has been frequently used as a painkiller and treatment for inflammatory diseases Yoon et al. (4). Son et al. (5) reported that more than 40 pharmaceutically active components are considered in bee venom, including peptides and enzymes, many of which have yet to be investigated (1). In several investigations, bee venom has been established to have anti-inflammatory and antibacterial characteristics. Pucca et al. (6) describe bee venom as comprising low molecular polypeptides and a complicated mixture of amino acids, peptides, proteins, enzymes, sugars, biogenic amines, volatile acids, phospholipids, and pheromones. In addition, the BV contains biologically active amines, such as histamine, adrenaline, and non-peptide derivatives, with a range of pharmacological properties. Melittin, apamin, and adolapin (polypeptides) act as anti-inflammatory and antibacterial factors and are the primary elements of BV (7). Melittin is the primary active component that enhances the pituitary and adrenal glands to produce catecholamine and cortisone, that has anti-inflammatory properties (8). Bee venom can be used safely and healthily in the rabbit industry instead of drugs (9). Melittin inclusion in the rabbits’ diets can increase their production efficiency (10) and could also be used for the prevention of disease (Apitherapy) (11). BV treatment provides a novel option for antimicrobial growth promoters in the nutrition of animals (11). On the other hand, the anti-oxidant, antibacterial, and analgesic properties of BV were also discovered by Zhang et al. (12). Moreover, because apamin and phospholipase A_2_ in BV have potent immuno-regulatory activity, several studies have demonstrated that BV therapy could help treat multiple immune disorders (13).

As a consequence of improper use, tetracycline residues may be detected in marketable animal products, representing a risk to humans and causing allergic reactions. Furthermore, prolonged use of low-dose antibiotics in products can contribute to the proliferation of drug-resistant microbes (14). As a result, BV significantly affects broiler performance and could be a feasible alternative to antimicrobial growth promoters to prevent the use of antimicrobials regularly (11).

For more than 60 years, antibiotics have been allowed to be used as growth promoters in poultry feeds without a prescription (15). Recently, many studies have been conducted to search for an alternative to antibiotics in the livestock industry. This ban is due to the fact that antibiotics as growth promoters in feed additives have led to the formation of resistant bacterial strains and antibiotic residues in animal products (16). From this perspective, medicinal plants can be used as an alternative to antibiotics and improve poultry live performance, such as extracted essential oils (17).

The modern trend in poultry feeding is to search for alternatives to antibiotics as growth promoters in poultry rations due to the harmful effects of antibiotic residues in poultry meat on human health. Most of the recent research tended to study bee venom to improve growth in broiler chicks, and there is a significant lack of studying the effect of bee venom on growing rabbits. This research investigated the impact of replacing BV instead of Oxytetracycline in growing rabbits’ drinking water on the productive performance, biochemical blood constituents, anti-oxidant status, immune response, and cecal microbial count of growing rabbits.

## 2. Materials and methods

In the current investigation, rabbits were handled under the recommendations of the Pharmaceutical and Fermentation Industries Development Center, City of Scientific Research and Technology Applications (SRTA-City), Alexandria, Egypt, with the approval of the Institutional Animal Care and Use Committees (IACUCs)/IACUC # 37-6F-1021.

### 2.1. Animals, housing environment, and experimental design

The current study was conducted at the experimental rabbitry farm in Qalubia governorate (the animals’ owner), Egypt, during the winter season (from January to March). Sixty healthy, hygienic male-growing Californian rabbits aged 35-day-olds with an initial body weight of (584 ± 9 g) were randomly assigned into five equivalent treatments (12 individuals each). The 1^st^ group received a basal diet and acted as a control group (C). The 2nd group received a basal diet +10 mg/kg body weight Oxytetracycline (OXT) in drinking water. The 3rd to 5th groups received a basal diet +2, 4, and 8 mg BV/kg body weight in drinking water, respectively (BV1, BV2, and BV3). BV and Oxytetracycline were delivered to the tested rabbits daily in drinking water for five experimental weeks.

In a controlled environment, rabbits were housed in galvanized wire cages with dimensions of approximately 60 × 55 × 40 cm and equipped with stainless steel nipples for drinking and feeders that monitored each rabbit’s feed intake. The temperature ranged from 18 to 25 degrees Celsius, relative humidity ranged from 45 to 58%, and the duration of daylight was 16 h. Feed and water were provided *ad libitum*.

The basal experimental diet was formulated and pelleted to cover the rabbits’ nutritional requirements, as shown in Table 1 (18). The doses of BV were suggested based on the doses used in adult chickens, pigs, and rabbits due to previous studies on BV on growth performance being very rare, and the majority was on broilers. In the morning, we weighed four rabbits from each treatment and calculated their average body weight, then dissolved an equivalent amount of BV (2, 4, and 8 mg BV/kg body weight) in 200 mL of water in the pan and, after the animal drank it we allowed them to drink from the nipples.

**Table 1:** Chemical composition and calculated analysis of the basal diet.

Ingredients	%
Yellow corn	6.22
Soybean meal, 44%	22.33
Wheat bran	23.33
Barley	15.00
Alfalfa hay	30.12
Ground limestone	1.00
Dicalcium Phosphate	1.20
Common salt	0.50
Vit. + min. Premix^*^	0.30
Total	100
Calculated analysis	
Crude protein, %	18.8
Crude fiber, %	13.0
Ether extract, %	3.0
Digestible energy (kcal/kg diet)	2,680
n-6 polyunsaturated FAs%	0.3
n-3 polyunsaturated FAs%	1.03
Zn, ingredients +Premix (50 + 50 mg)	100
Determined analysis (g/kg diet)	
Dry matter	897.1
Organic matter	801.4
Crude protein	169.8
Crude fiber	138.5
Ether extract	26.2
Nitrogen-free extract	575.0
Ash	87.9

*Each kg of mixture premix contained: Vit. A 10,000,000 IU; vit. D3 3,000,000 IU; vit. E 2500 Mg; vit. K3 4000Mg; vit. B1 5000Mg; vit. B2 500Mg; vit. B6 2500Mg; vit. B12 5 Mg; vit. C 10,000 Mg; nicotinamide 20,000 Mg; pantothenic acid 5,000 Mg; biotin 5 Mg; folic acid 250 Mg; D.L methionine 10,000 Mg; L. lysine 5,000 Mg; Fe 70 Mg; CuSO_4_ 120 Mg; ZnSO_4_ 60 Mg; MnSO_4_ 160 Mg; CoSO_4_ 16.2 Mg; MgSO_4_ 70 Mg; calcium iodate 50 Mg; NaCl 7,000 Mg and KCl 15,000 Mg.

### 2.2. Bee venom source and its chemical analysis

The bee venom (BV) experimental sample was collected from El-Beshkar Manhal for Bee breeding and Bee products in Egypt. BV was collected manually or following the electric method described by Han et al. (11). Both samples were subjected to chemical analysis according to the method described by Hind et al. (19). Oxytetracycline (OXT; 20%) was produced by the Arab Co. for Gelatin and Pharmaceutical products in Egypt.

### 2.3. Data collected

#### 2.3.1. Productive performance

Live body weight (LBW) was individually recorded for each treatment at the start time as initial live body weight and the end of the 1st, 2nd, 3rd, 4th, and 5th week. Each rabbit’s body weight gain (BWG; as grams) was recorded as follows:

BWG = live body weight at the current week - live body weight at the previous week.

Also, weekly feed intake (FI) was recorded for each animal of each treatment (as g feed/rabbit), and feed conversion ratio (FCR; as grams of feed intake per gram of body weight gain) was calculated.

#### 2.3.2. Carcass characteristics

At the end of the growth experiment, six rabbits per treatment were selected and slaughtered to assess carcass weight relative to live body weight. Total edible parts were calculated. Total edible parts% = carcass+ kidney+ heart+ liver (20).

#### 2.3.3. Digestibility experiment

The digestibility values of dry matter (DM), organic matter (OM), crude protein (CP), crude fiber (*CF*), ether extract (EE), nitrogen-free extract (NFE), and digestible crude protein (DCP) were determined at the end of the growth experiment. Three animals from each group were confined in metabolic cages with a stainless-steel screen with 4 mm mesh to keep feces while allowing urine to pass unhindered. The digestion experiment and collection period lasted 5 days, during which feed intake was registered. Feces were collected daily before breakfast, weighed fresh, treated with 2% boric acid to catch any ammonia produced from feces, and dried in an air-drying oven at 60°C for 24 h. Afterwards, the feces were crushed and mixed before being stored for chemical analysis (21). The experimental diets’ digestibility coefficients and nutritional values were determined using chemical analysis of feed and feces samples.

#### 2.3.4. Blood sampling and blood biochemical constituents

At the end of the experiment period, blood samples of each treatment were collected from 6 rabbits (from marginal ear veins) of each treatment before providing feed and water in the morning. Heparin was used as an anticoagulant, but some samples were without heparin to obtain serum. Plasma or serum was obtained by centrifugation of the blood samples at 3500 rpm for 20 min and stored at −20°C for later biochemical analysis.

Plasma Total protein (TP), Albumin (Albu), and Globulin (Glob) concentration as (g/dl) were measured. Plasma Total cholesterol (TChol), High-density lipoproteins (HDL) cholesterol, Low-density lipoproteins (LDL) cholesterol, and Triglycerides (Trgly) concentrations as (mg/dl) were estimated. Liver enzymes activity (aspartate aminotransferase (AST) and alanine aminotransferase (ALT)) were assessed. Total anti-oxidant capacity (TAC) as (mmol/L) and Malondialdehyde (MDA) as (μmol/ml) were determined. Glutathione peroxidase (GPx), superoxide dismutase (SOD), and catalase enzyme activities were determined.

#### 2.3.5. Cecal microflora count

The carcasses were subsequently opened, and the entire intestinal tract was removed aseptically. The tract was then divided into sections that were ligated with light twine before separating the ceca from the tract intestine and then stored in sterile bags filled with ice-cold cryoprotective broth (50 mL), which was used to keep gut flora alive (22) and stored at −80°. The cecal digesta was thawed and aseptically emptied in a new sterile bag for bacterial enumeration. Immediately cecal digesta was diluted to 10-fold (i.e., 10% w/v) with sterile ice-cold anoxic phosphate-buffered saline (PBS) (0.1 M, pH 7.0) and subsequently homogenized for 3 min. Each cecal homogenate was serially diluted from 10–^1^ to 10–^7^. Dilutions were subsequently plated in duplicate on selective agar media for target bacterial groups, and the enumeration results were expressed as colony-forming units (cfu) log ^10^ per ml.

The total count of anaerobic bacteria and *Escherichia coli* (*E. coli*) was determined using the method outlined by Üollins-Racie (23), and the lactobacilli bacteria count was evaluated following the method described by Kim and Goepfert (24).

### 2.4. Statistical analysis

Data collected were tabulated and statistically analyzed using the one-way ANOVA of SAS (25). Significances of the effects were tested at levels *p* ≤ 0.05 (*) and *p* ≤ 0.01 (**) and compared using the Turkey test (25).

## 3. Results

### 3.1. Chemical analysis of Egyptian bee venom

Chemical analysis of BV showed that the main components were melittin, protease, hyaluronidase, and phospholipase A2 (Table 2). The electrical method was more effective in collecting BV with more highly concentrated active ingredients than the manual method.

**Table 2:** The effect of the BV collection method on its content from active ingredients.

Methods	Honey bee venom analysis			
	Melittin lysis (%)	Protease (U/mg)	Hyaluronidase (U/mg)	Phospholipase A2 activity (U/mg)
Manual methods	46.71	32,08	99.37	102.77
Electrical methods	67.44	135.08	129.81	222.38
SEM	0.24	0.37	0.28	0.38

### 3.2. Impact of BV on productive performance

Data clarified that adding OXT or different doses of BV to growing rabbits’ drinking water resulted in a significant increase (*p* ≤ 0.01) in LBW and BWG in contrast to the control group Table 3. Furthermore, BV groups showed higher LBW and BWG than the OXT group, with a preference for the BV2 dose. From Table 3, BV rabbits groups consumed less feed than the control and OXT groups, and this effect was significant (*p* ≤ 0.01) in comparison to the control group only. FCR of groups treated with BV showed a significant improvement (*p* ≤ 0.01) relative to the control and OXT groups.

**Table 3:** Impact of adding BV to the drinking water of growing rabbits on productive performance.

Items	Treatments					SEM	*P*-value
	C	OXT	BV1	BV2	BV3		
Initial BW (g)	583	585	589	581	584	9.156	NS
Final BW (g)	1974^b^	2014^a^	2089^a^	2160^a^	2138^a^	33.406	0.0091
BWG (g/rabbit)	1396^b^	1427^ab^	1520^a^	1578^a^	1556^a^	32.772	0.0094
FI (g/rabbit)	5552^a^	5495^ab^	5522^b^	5338^b^	5290^b^	15.003	0.0001
FCR (g feed/g BWG)	3.97^a^	3.89^a^	3.52^b^	3.40^b^	3.45^b^	0.079	0.0002

^a,b^Values in the same row with different superscripts differ significantly (*p* ≤ 0.05).

### 3.3. Impact of BV on nutrient digestibility

Table 4 illustrates the data of nutrient digestibility (DM, CP, EE, *CF*, and NFE) and (TDN, DCP, and DE) of growing rabbits due to adding different BV doses to their drinking water. Notably, the oral administration of OXT and BV to growing rabbits resulted in an improvement in digestibility coefficients (CP, EE, NFE, and *CF*) compared to those of the control group, and this improvement was significant (*p* ≤ 0.01) with the low dosage of BV, which had similar results to TOX. BV doses and OXT also significantly increased the nutritive digestibility values (TDN, DCP, and DE).

**Table 4:** Impacts of adding BV to the drinking water of growing rabbits on digestibility coefficient and nutritive values.

Items	Treatments					SEM	*P*-value
	C	OXT	BV1	BV2	BV3		
Digestion coefficient (%)							
DM	71.92	73.15	73. 06	72.45	72.56	0.87	0.093
CP	59.61^b^	68.43^a^	69.06^a^	64.79^ab^	67.67^a^	1.22	0.006
EE	58.97^b^	67.92^a^	65.80^a^	64.27^a^	63.68^ab^	1.00	0.003
NFE	72.62	77.23	77.84	73.61	72.23	0.54	0.083
*CF*	44.51^b^	54.21^a^	54.91^a^	47.77^b^	48.24^b^	0.75	0.002
Nutritive values							
TDN, %	61.36^b^	69.41^a^	69.24^a^	64.68^b^	62.92^b^	0.90	0.007
DCP, %	10.25^b^	12.42^a^	13.15^a^	12.01^a^	12.56^a^	0.05	0.001
DEE (kcal/kg)	2718^b^	3074^a^	3067^a^	2865^b^	2787^b^	3.75	0.008

^a,b^Values in the same row with different superscripts differ significantly (*P* ≤ 0.05). TDN, % = DCP % + DCF % + DEE % (2.25) + DNFE %; DCP, % = Digestibility coefficient of CP × CP% of the diet; DEE (kcal/kg) = TDN × 44.3.

### 3.4. Impact of BV on carcass traits and some organs’ weight

Table 5 presents data on carcass characteristics and the effect of BV treatment on treated rabbits could be noticed. Generally, treated growing rabbits with OXT or BV at different dosages significantly boosted (*p* ≤ 0.01) the percentage of carcass weight and the total edible parts percentage in comparison to the control group. In addition, BV treatments had a non-significant higher carcass and total edible parts values than the OXT treatment with a preference for BV2 dosage (4 mg BV/kg BW). Data in Table 4 illustrated that the percentage of the relative weight of the small intestine, cecum (without emptying the internal contents), pancreas, and abdominal fat for treated growing rabbits increased significantly (*p* ≤ 0.01) in both OXT and BV groups compared with the control, while the BV2 dosage had the highest values. On the other hand, the liver, heart, and spleen weights were not affected by OXT or BV treatments, while the kidney weight was decreased.

**Table 5:** Impact of adding BV to the drinking water of growing rabbits on carcass traits and some organs’ weight.

Items	Treatments					SEM	*P*-value
	C	OXT	BV1	BV2	BV3		
Hot carcass %	52.1^b^	57.87^a^	59.79^a^	60.85^a^	59.11^a^	6.533	0.0046
Liver, %	5.69	5.85	5.47	5.97	5.66	0.748	0.1077
Heart, %	0.53	0.50	0.57	0.55	0.48	1.347	0.4056
Kidneys, %	1.12^a^	0.84^b^	0.81^b^	0.83^b^	0.98^ab^	0.039	0.010
Total edible parts %	59.44^b^	67.21 ^a^	67.47^a^	69.03^a^	67.91^a^	0.727	0.0464
Pancreas	0.69^b^	0.79^ab^	0.77^ab^	0.86^a^	0.72^ab^	0.018	0.0001
Spleen	0.11	0.11	0.09	0.08	0.09	0.001	0.0613
Small intestine	4.93^b^	6.21^ab^	7.07^a^	7.99^a^	8.12^a^	0.301	0.0032
Cecum	7.78^b^	9.61^ab^	11.08^a^	11.07^a^	9.76^ab^	0.372	0.0048
Abdominal fat	1.59^b^	3.48^a^	2.08^b^	2.89^a^	2.91^a^	0.102	0.0001

^a,b^Values in the same row with different superscripts differ significantly (*P* ≤ 0.05).

### 3.5. Impact of BV on blood biochemical constituents

From Table 6, it could be summarized that treated growing rabbits with BV at any studied doses increased plasma TP and Glob concentration significantly (*p* ≤ 0.01) comparing to the control or OXT treatment. In contrast, the plasma Albu concentration did not differ from the reference groups. Within the same context, plasma Glob concentration was increased due to treating growing rabbits with BV, and this increase was significant compared to the control group only. AST and ALT (liver function enzymes) activities decreased significantly in BV-treated growing rabbits (*p* ≤ 0.01) than the untreated group or that treated with OXT. Blood TChol and Trgly concentrations were gradually and significantly reduced in BV-treated groups in comparison with the OXT or the control groups, and this decrease was BV dose-dependent. Both O.X.T. and BV treatment resulted in a significant reduction (*p* ≤ 0.01) in the harmful part of cholesterol (LDL and VLDL) concentration relative to the control group; this reduction was BV dose-dependent manner.

**Table 6:** Impact of adding BV to the drinking water of growing rabbits on blood biochemical constituents.

Items	Treatments					SEM	*P*-value
	C	OXT	BV1	BV2	BV3		
TP (g/dL)	5.50^c^	6.50^b^	6.93^ab^	7.20^a^	7.19^a^	0.081	0.0001
Albu (g/dl)	4.10	3.40	3.90	4.10	3.75	0.146	0.316
Glob (g/dl)	1.04^b^	3.10^a^	3.03^a^	3.10^a^	3.44^a^	0.156	0.0038
AST (U/L)	35.99^a^	27.90^b^	23.90^b^	23.93^b^	24.50^b^	0.649	0.0001
ALT (U/L)	42.08^a^	39.98^a^	26.67^b^	24.08^b^	23.00^b^	0.350	0.002
Trgly (mg/dL)	157.50^a^	117.33^b^	100.00^bc^	92.00^c^	90.33^c^	2.481	0.0001
TChol(mg/dL)	80.33^a^	73.00^b^	65.33^c^	60.67^c^	59.33^c^	0.798	0.0001
HDL-c (mg/dL)	34.68	38.67	39.97	38.33	36.33	1.144	0.519
LDL-c (mg/dL)	14.18^a^	10.87^b^	5.36^c^	4.60^c^	4.27^c^	1.537	0.049
VLDL-c (mg/dL)	31.50^a^	23.47^b^	20.00^bc^	18.40^c^	18.07^c^	0.496	0.0001

^a,b,c^Values in the same row with different superscripts differ significantly (*P* ≤ 0.05).

### 3.6. Impact of BV on immune response and anti-oxidant status

Table 7 summarizes the data on the effect of treating growing rabbits with BV on the anti-oxidant status and immune response. The results illustrated a gradual and highly significant increase in blood TAC levels and anti-oxidant enzyme activities (S.O.D., GPx, and CAT) in the groups treated with BV in comparison to the reference groups (control and OXT groups). In contrast, blood MAD level was significantly decreased. Furthermore, adding OXT to rabbitsˊ drinking water resulted in a significant malicious effect on blood anti-oxidant properties, such as TAC level and S.O.D., GPx, and CAT enzymes activities that significantly decreased compared to the control group. Treating growing rabbits with BV enhanced the immune system, which could be noticed through the increase of IgM and IgG levels compared to the control or OXT groups.

**Table 7:** Impact of adding BV to the drinking water of growing rabbits on anti-oxidant status and immunoglobulin response.

Items	Treatments					SEM	*P*-value
	C	OXT	BV1	BV2	BV3		
TAC (μmol/ml)	1.91^c^	0.54^d^	2.42^b^	2.68^b^	3.01^a^	0.028	0.0001
MDA (nmol/ml)	0.42^b^	0.56^a^	0.33^c^	0.28^c^	0.29^c^	0.04	0.0001
SOD (IU /ml)	6.38^b^	3.43^c^	7.32^a^	8.41^a^	7.8^a^	0.54	0.0001
GPx (IU /ml)	6.09^b^	2.23^c^	7.84^a^	8.12^a^	8.56^a^	1.05	0.003
CAT (IU /ml)	71.16^b^	48.65^c^	87.75^a^	83.43^a^	89.77^a^	3.134	0.0004
IgG (mg/dl)	331^d^	475^cb^	645^a^	525^b^	415^c^	40.81	0.0001
IgM (mg/dl)	35.00	39.00	53.00	43.00	40.00	4.209	0.065

^a,b,c,d^Values in the same row with different superscripts differ significantly (*P* ≤ 0.05).

### 3.7. Impact of BV on caecum microbial count

Table 8 illustrates the results of caecum microbial count (log^10^ (cfu/g)) due to adding BV in rabbits drinking water. The data showed that BV, like OXT, led to a decrease in cecal TBC, which was BV-dose dependent, whereas this effect was significant with BV low dosage only and decreased with increasing BV dosage. Likewise, pathogenic bacterial count (*Salmonella* spp., *E.coli* spp., *Proteus* spp., and *Clostridia* spp.) were significantly reduced due to adding OXT to growing rabbits drinking water or BV, and the low BV dosage showed a similar effect to that of the OXT dosage.

**Table 8:** Impact of adding BV to the drinking water of growing rabbits on cecum bacterial count.

Items	Treatments					SEM	*P*-value
	C	OXT	BV1	BV2	BV3		
TBC, 10^6^/Cmm	3.00^a^	2.5b^c^	2.4^c^	2.7^ab^	2.85^a^	0.08	0.001
Salmonella spp. 10^5^/Cmm	0.81^a^	0.53^d^	0.63^c^	0.72^b^	0.73^b^	0.21	0.001
E.coli spp. 10^5^/Cmm	2.6^a^	2.15^b^	1.75^c^	2.00^b^	2.2^b^	0.34	0.001
Proteus spp. 10^5^/Cmm	1.21^a^	0.54^d^	0.57^d^	0.86^c^	0.96^b^	0.09	0.001
Clostridia spp. 10^5^/Cmm	1.85^a^	1.75^a^	1.5^b^	1.45^b^	1.55^b^	0.05	0.001

^a,b^Values in the same row with different superscripts differ significantly (*P* ≤ 0.05).

## 4. Discussion

Regarding the BV collection method, it can be summarized that the electric method is more effective than the manual method, as the active ingredients were present in a higher concentration in BV from the electric technique than the manual method. In our study, the primary substances in BV were melittin, protease, hyaluronidase, and phospholipase A2; this result was in accordance with Teoh et al. (26) and Mammadova and Topchiyeva (27). According to Damianoglou et al. (28), approximately 60 components have been identified in bee venom, with melittin being the most abundant. In the same context, several studies mentioned that Honey bee venom comprises many compounds, including enzymes, proteins, peptides, and numerous other tiny substances (amino acids, catecholamines, sugars, and minerals) (29). According to El-Hanoun et al. (9), the active substance in BV may be a significant external environmental component that can directly impact animal welfare.

Increased daily BWG and decreased daily FI of BV rabbits’ groups reflected in FCR that was significantly improved (*p* ≤ 0.01) versus the control and OXT groups. A previous study by Han et al. (11) demonstrated that at 28 days of age, adding BV to broiler drinking water significantly increased BWG compared to the control group (*p* < 0.05); as well, FI was numerically higher but without significance. So, BV can potentially replace antimicrobial growth promoters in broiler nutrition. This is in agreement with Han et al. (30), who indicated BV injection resulted in a net increase in BW gain and survivability in piglets. According to Rabie et al. (10) and El-Hanoun et al. (9), BV substance can stimulate productive performance, which could be ascribed to its health benefits, including the prevention/treatment of diseases (Apitherapy) (8). Additionally, it has antimicrobial properties (11). Likewise, stinging or subcutaneously injecting piglets with BV increased LBW significantly with decreased FI. Moreover, BV improved the survivability of the piglets, so it was possible to use BV as an alternative to antibiotics growth promoters (30, 31). In contrast, Ahmad et al. (32) stated that the FI of birds supplemented with BV was significantly higher than that of control birds. Still, this increase was positively correlated with the increasing BWG of the treated birds. On the other hand, injecting broilers with BV extract did not affect LBW at 42 days and BWG from 0 to 6 weeks; additionally, FI was not significantly lower in the treated groups than in the control group (33). Moreover, according to Lu et al. (34), melittin does not harm the immune system, which should be another contributing factor to growth performance in a highly concentrated production environment in the broiler industry. It seems likely that BV strengthens the immune response to the normal environmental, social, and nutritional challenges faced by newly born chicks.

Carcass characteristics (Hot carcass and Total edible parts) improvement that was noticed in the BV treated groups was attributed to the improvement that happened in the growth performance of BV rabbits compared to the control group, whereas BWG was significantly increased and FI significantly decreased, which in return was reflected on FCR improvement, which referred to BV boosted rabbit ability to convert feed to meat. On the other hand, the data presented in Table 4 illustrated an increase in the digestibility Coefficient and Nutritive Values for BV groups in comparison to the control group. These results indicated increased nutritive absorption values through the intestine; consequently, there was an abundance of nutrients to build muscles. This hypothesis is consistent with the increased small intestine weight in the BV groups in contrast to the control groups. In the same context, the increased pancreas weight of BV rabbits more than the control group indicates an increase in pancreatic function of insulin hormone secretion, which plays a vital role in carbohydrate metabolism.

Moreover, increased abdominal fat relative weight and decreased blood Trgly concentration of BV treated groups relative to the control group can be explained by the fact that BV affected lipid metabolism and stimulated and enhanced the lipid transfer process from blood to be stored in the adipose tissue. Our results agree with that found by El-Kholy (35), who reported that the significant improvement in carcass characteristics for treated groups could be due to the treated rabbits’ improved growth performance, which mirrors the rabbits’ health. Furthermore, Lu et al. (34) mentioned that melittin contributed to growth performance in intensive broiler production systems. Besides, the BV boosted an immune response to encounter the challenges of the newly born chicks’ normal environmental and nutritional conditions. Also, BV supplementation counterbalanced stress within days after birth. In addition, no morphological injury signs to the studied organs were observed under BV treatment, which was consistent with that found in broilers treated with BV in drinking water (11).

Increasing plasma TP in the present study agreed with that obtained by El-Hanoun et al. (9) and Elkomy et al. (36), who demonstrated that injected adult male and female rabbits with BV significantly increased blood TP and this effect was dependent on BV dosage (37). BV induced the immune system to increase plasma Total Glob concentration and immune-globulin fractions (IgG and IgM), which improved rabbit health. Our results were consistent with those of Elkomy et al. (36), El-Hanoun et al. (9), and Gurafi (37), who stated that the BV treatment increased plasma Glob levels significantly more than the control group. This increase may be attributable to the activation of the immune system due to the antigenic properties of BV’s active peptide content (38). In contrast, Ali and Mohanny (33) showed that injecting broiler chicks with BV extract had an insignificant impact on total protein, albumin, and globulin. Likewise, Han et al. (11) mentioned that significant impacts on total protein, albumin, and globulin were noticed due to adding BV via drinking water to chicks. The reduction in AST and ALT enzymes activities that happened in our study following the findings of El-Hanoun et al. (9) and Elkomy et al. (36), who had a similar result when injected subcutaneously with male and female adult rabbits with BV, and this effect revealed an improvement in liver function. Opposite results were found by Han et al. (11); no significant impacts on AST and ALT were observed in broilers receiving BV in their drinking water. In addition, Bolarinwa et al. (1) demonstrated that stinging with BV had no effect on AST and ALT levels when comparing the control and investigated treatments. These previous findings suggest that the impact of BV on the AST and ALT enzyme activities may be dose-dependent, and a high BV dose is needed to decrease the activity of liver enzymes.

Our results revealed that TChol and the harmful part of Chol (LDL and VLDL Chol) were significantly decreased, and the beneficial Chol (HDL Chol) was increased, as well, plasma Trgly level was decreased significantly and this decrease was associated with increased abdominal fat deposition. Decreasing TChol and Trgly levels described in the current study are consistent with El-Hanoun et al. (9), who mentioned that injecting rabbit’s male subcutaneously with BV gradually and significantly decreased plasma Total lipids and TChol. In the same context, BV decreased plasma TChol and Trgly content in mice (39). The same result was found by Mousavi et al. (40) in diabetic rats and revealed that BV significantly reduced the elevated levels of Trgly and TChol to normal levels. In contrast, Han et al. (11) illustrated that BV supplementation via drinking water had no significant impacts on TChol. The relationship between abdominal fat and plasma lipids could be explained by Whitehead and Griffin (41), they found that body fat content correlated closely with plasma VLDL in addition to LDL triglycerides, and it could be used this relationship for selecting leanness in broiler breeding programmers on the basis of measurements of plasma VLDL plus LDL triglyceride. Wayne et al. (42) reported that abdominal fat and blood plasma triglycerides were significantly correlated with body fat. These correlation coefficients indicated that a different relationship exists between fatness and plasma triglyceride concentration in male and female turkeys.

The present results of the effect of BV on redox status agree with that of Elkomy et al. (36), who studied the impact of different BV doses on TAC, GSH, GST, GPx, and SOD. He also found a gradual and significant rise in TAC and anti-oxidative enzyme activities versus the control group, while MDA and TBARS were significantly decreased. Increased anti-oxidant levels due to BV treatment increased the examined does’ resistance to biological and environmental stresses. In the same context, the findings of El-Hanoun et al. (9) on male rabbits, Kim et al. (43), and Han et al. (11) on broiler chickens revealed that BV increased the anti-oxidant profile. According to the findings of Crott and Fenech (44) and Kim et al. (43), BV possesses pharmacological anti-oxidant properties and can protect against cellular injury, thereby reducing oxidative damage to lipids, proteins, and DNA. Kocyigit et al. (45) found a significant increase in total anti-oxidant status (TAS), total oxidant status (TOS), and oxidative stress index (OSI) in rats treated with BV. From previous studies, the SOD, CAT, and GPx are essential enzymes that play an endogenous anti-oxidant protection system and play a crucial part in removing free radicals and maintaining the intracellular redox balance (46). El-Speiy et al. (47) stated that the rise in GPx and SOD activity was indicative of improving anti-oxidant capacity. Furthermore, blood MDA concentration was used as an indicator of lipid peroxidation in the body (48); consequently, the stage of cell injury and lipid peroxidation can be determined by measuring the concentration of MDA, which are the principal products of polyunsaturated lipid peroxidation (46). Based on the previous mentions, the improved TAC, GPx, CAT, and SOD components in the BV groups reflect the fact that BV has anti-oxidant properties that enhance the anti-oxidant defense against oxidative damage, resulting in a decrease in lipid peroxidation markers such as MDA concentration.

Data revealed that BV boosted IgM and IgG levels, and the low-dose BV had the main effect on alerting the immune system through the impact of BV on immuno-globulin fractions, which decreased with the increase of BV dosage. Our findings align with prior research by Elkomy et al. (36) in rabbit does and El-Hanoun et al. (9) in rabbit bucks; they reported that injecting BV led to elevate IgG, IgA, and IgG levels significantly. According to Ali and Mohanny (33), BV as a natural product could stimulate and improve broiler chickens’ immune response without adversely affecting productive performance, possibly because of superoxide production (11). Depending on its pharmacological components, BV can boost the immune system of animals, including cortisone production and antibody creation, and influence cytokine production (5, 49, 50). Bee venom is a blend of biochemicals ranging from small peptides and enzymes to biogenic amines. It is capable of triggering severe immunologic reactions owing to its allergenic fraction. Venom components are presented to the T cells by antigen-presenting cells within the skin. These Th2 type T cells then release IL-4 and IL-13, which subsequently direct B cells to class switch to the production of IgE. BV can induce elevation of specific IgE and IgG antibodies (51) and leads to the production of IgE antibodies, which can respond to various antigens (52).

Data on cecum microbial count and differentiation agreed with the previously detected results. Previous studies have mentioned that BV is a mixture of substances and has bioactive properties, including growth promotor (31), anti-inflammatory (53), and antimicrobial (11) in treated animals with no side effects detected on them. According to Leandro et al. (54), the BV antimicrobial activity may be due to several peptides, which involve melittin, apamin, adolapin, enzymes, biologically-active amines, and non-peptide parts. Above that, BV contains Phospholipase A2 (PLA2), which has antibacterial properties, plus mellitin contributes to this antibacterial effect (55, 56). The same result was reported by Dani et al. (57) and Permual et al. (58), who noted that Mellitin is a primary component of BV and has antibacterial properties. The data obtained by Zolfagharian et al. (59) showed that BV inhibited bacterial growth and had stronger antibacterial efficacy than the gentamicin antibiotic. Consequently, BV has antimicrobial properties against gram-positive as well as gram-negative bacteria, including *Escherichia coli* (*E. coli*), *Salmonella* spp., *Enterobacter cloacae*, *Staphylococcus aureus* (*S. aureus*), and coagulase-negative *Staphylococcus* (60). Moreover, numerous impacts on distinct cell types involving antiviral and anti-inflammatory effects have been observed (59). Ortel and Markwardt (61) also mentioned that microorganisms were also found to be more sensitive to BV at lesser concentrations.

## 5. Conclusion

In conclusion, bee venom (BV) at 2, 4, and 8 mg dosages led to a significant increase in LBW and BWG accompanied by a decrease in FI, resulting in improved FCR and carcass traits. Moreover, BV positively affects the immunological and anti-oxidative responses and reduces the pathogenic bacteria count in the hindgut in growing rabbits.

Bee venom can be an effective, safe, and healthy alternative to antibiotics for use in rabbit farms to improve the productivity and health status of growing rabbits and reduce the activity of pathogenic bacteria in the cecum.

## Data availability statement

The original contributions presented in the study are included in the article/supplementary material. Further inquiries can be directed to the corresponding authors.

## Ethics statement

The animal studies were approved by Animals were handled in the present study following the guidelines of the Pharmaceutical and Fermentation Industries Development Center, City of Scientific Research and Technology Applications (SRTA-City), Alexandria, Egypt, after the approval of the Institutional Animal Care and Use Committees (IACUCs)/IACUC # 37-6F-1021. The studies were conducted in accordance with the local legislation and institutional requirements. Written informed consent was obtained from the owners for the participation of their animals in this study.

## Author contributions

AE, ME-S, SH, and TS: supervision, project administration, conceptualization, methodology, formal analysis, supervision, and writing–original draft. ME-B: resources. AE, HaE, and TS: conceptualization, methodology, formal analysis, and investigation. MW, SC, IK, and HoE: supervision, visualization, data curation, and writing–review and editing. All authors contributed to the article and approved the submitted version.

## Funding

This research was supported by Basic Science Research Program through the National Research Foundation of Korea (NRF) funded by the Ministry of Education (NRF-RS-2023-00275307).
